# Up-skilling the next generation of researchers to analyse complex linked population-level datasets: ADR UK PhD studentships case study

**DOI:** 10.23889/ijpds.v9i5.2536

**Published:** 2024-09-18

**Authors:** Saba Mir, Emily Oliver, Emma Gordon

**Affiliations:** 1 UK Research and Innovation

## Objectives

Homelessness is a complex and pressing issue affecting individuals and communities worldwide, including Northern Ireland. Understanding its root causes and associated risk factors is crucial for effective interventions. This study aims to identify primary contributors to homelessness and explore risk factors linked to chronic homelessness in Northern Ireland.

## Methods

Utilizing an anonymized, linked dataset incorporating Northern Ireland Housing Executive (NIHE) and health and social care data, we will conduct a pioneering record linkage study. This analysis will focus on unravelling homelessness dynamics, particularly chronic homelessness, spanning from 2012 to 2022.

## Results

Through our comprehensive analysis of using linked administrative datasets, we anticipate unveiling significant insights into the prevalence and persistence of chronic homelessness in Northern Ireland. By employing descriptive analyses, we will uncover nuanced patterns and trends, shedding light on the multifaceted dynamics of homelessness within the region. Moreover, our examination aims to identify key factors contributing to the persistence of chronic homelessness, providing crucial insights for developing targeted interventions and support systems. The outcomes of this analysis will not only contribute to a deeper understanding of the complexities surrounding homelessness but also serve as a foundation for evidence-based policymaking and community initiatives aimed at alleviating homelessness in Northern Ireland.

## Conclusion

This study has the potential to increase our understanding about the characteristics, needs and outcomes of the homeless population in Northern Ireland. It is possible that the results may contribute to facilitating further cross-departmental integration and commitment to addressing vulnerable homeless households with complex needs.

